# Dual Inhibition of *Salmonella enterica* and *Clostridium perfringens* by New Probiotic Candidates Isolated from Chicken Intestinal Mucosa

**DOI:** 10.3390/microorganisms9010166

**Published:** 2021-01-13

**Authors:** Ayesha Lone, Walid Mottawea, Yasmina Ait Chait, Riadh Hammami

**Affiliations:** 1 NuGUT Research Platform, School of Nutrition Sciences, Faculty of Health Sciences, University of Ottawa, Ottawa, ON K1H8M5, Canada; alone069@uottawa.ca (A.L.); wmott020@uottawa.ca (W.M.); yaitc043@uottawa.ca (Y.A.C.); 2 Department of Microbiology and Immunology, Faculty of Pharmacy, Mansoura University, Mansoura 35516, Egypt

**Keywords:** poultry, Lactobacilli, probiotic candidates, antimicrobial activity, bacteriocins, *Salmonella*, *Clostridium perfringens*

## Abstract

The poultry industry is the fastest-growing agricultural sector globally. With poultry meat being economical and in high demand, the end product’s safety is of importance. Globally, governments are coming together to ban the use of antibiotics as prophylaxis and for growth promotion in poultry. *Salmonella* and *Clostridium perfringens* are two leading pathogens that cause foodborne illnesses and are linked explicitly to poultry products. Furthermore, numerous outbreaks occur every year. A substitute for antibiotics is required by the industry to maintain the same productivity level and, hence, profits. We aimed to isolate and identify potential probiotic strains from the ceca mucosa of the chicken intestinal tract with bacteriocinogenic properties. We were able to isolate multiple and diverse strains, including a new uncultured bacterium, with inhibitory activity against *Salmonella* Typhimurium ATCC 14028, *Salmonella* Abony NCTC 6017, *Salmonella* Choleraesuis ATCC 10708, *Clostridium perfringens* ATCC 13124, and *Escherichia coli* ATCC 25922. The five most potent strains were further characterized for their probiotic potential (i.e., sensitivity to antibiotics and tolerance to gastrointestinal physicochemical conditions). Our analyzed lactobacilli strains exhibited some interesting probiotic features while being inhibitory against targeted pathogens.

## 1. Introduction

*Salmonella* is one of the leading causes of foodborne diseases worldwide, followed closely by *Clostridium perfringens* [1,2]. In Europe, one in four human infections by *Salmonella* shows resistance to three or more antimicrobials that are routinely used in human and animal medicine [3]. In the US, 5% of tested *Salmonella* was found to be resistant to five or more types of antibiotics in 2011, and, by the end of 2018, the number of *Salmonella* outbreaks in the US was sitting at fourteen. Five were linked to chickens, raw chickens, and eggs, which was twice as many as seen in 2015 and 2016 [4,5]. As for *C. perfringens*, the CDC estimates that it causes 1 million cases of foodborne illnesses per year with a substantial economic loss to the poultry industry due to the high rate of poultry mortality [6,7].

Exposure to chicken and other poultry products has been identified as a common source of both campylobacteriosis and salmonellosis outbreaks and as a risk factor for sporadic infection with these pathogens [8]. With the recent ban on the use of antibiotics as growth promoters and for prophylaxis [9,10], producers will have to change the way the industry raises broilers by trying to mitigate the spread of poultry pathogens while maintaining the productivity of the past. Proposed alternatives to antibiotic use in poultry feeds include phytogenic feed additives, feed acidifiers, antimicrobial peptides, bacteriophages, antibodies, prebiotics, and probiotics [11,12]. Probiotics are promising alternative growth promoters, and evidence of their beneficial effects is accumulating in poultry production [13]. The mode of action of probiotic feed additives in poultry is mainly based on four principles: (i) maintaining normal intestinal microbiota by competitive exclusion and antagonism [14], (ii) altering metabolism by increasing digestive enzyme activity, and decreasing bacterial enzyme activity and ammonia production [15], (iii) improving feed consumption and digestion [16], and (iv) stimulating the immune system [17]. Furthermore, they exert indirect antimicrobial effects against pathogenic bacteria, which minimize the possibility of developing antimicrobial resistance among the bird commensals, while enhancing the proliferation of beneficial microbes [12]. Particularly, bacteriocins and their producing probiotics, such as *Enterococcus* spp. and some *E. coli* strains, have acquired great attention as natural antibiotic alternatives in the food industry [17,18,19]. However, the prevalence of antimicrobial resistance genes (ARGs) and virulence factors among the investigated strains hinder their application in the food industry [18]. While many studies exist that show beneficial effects of such probiotics in chickens, in vitro, in vivo, or both, the results have been inconsistent [20]. Broad spectrum probiotics isolated from chicken indigenous microbiota with dual activity against *Salmonella enterica* and *Clostridium perfringens* are still unidentified. In this study, we aimed to isolate potential probiotic candidates from the ceca mucosa of broiler chicken that can control the growth of common poultry pathogens including *Salmonella enterica* and *Clostridium perfringens*.

## 2. Materials and Methods

### 2.1. Bacterial Strains and Culture Conditions

Indicator strains of *Salmonella enterica* serovar Abony NCTC 6017, *Salmonella enterica* serovar Typhimurium ATCC 14028, and *Salmonella enterica* serovar Choleraesuis ATCC 10708 were cultured in de Man, Rogosa and Sharpe (MRS; Criterion, Hardy Diagnostics, CA, USA) broth and incubated aerobically at 37 °C for 24 h. *Clostridium perfringens* ATCC 13124 was cultured in fastidious anaerobic broth (LAB M, Heywood, UK) supplemented with (0.5%) yeast extract and incubated anaerobically for 24 h at 37 °C. Isolated strains from this study were cultured in MRS broth supplemented with (0.1%) l-cysteine for 24 h at 37 °C under aerobic or anaerobic (a gas mixture of 5% CO_2_, 5% H_2_, and 90% N_2_, Whitley Anaerobe Systems A35, Don Whitley Scientific, Bingley, UK) conditions. All isolated strains were purified three times on respective agar media plates. The strains were maintained in 25% glycerol stock at −80 °C until use.

### 2.2. Preparation of Ceca Mucosal Scrapings from the Chicken Intestinal Tract

Broiler chicken intestinal tracts were obtained from a local commercial slaughterhouse (Berube Poultry, Mountain, Canada) and Ringers solution (NaCl 7.2 g/L, CaCl_2_ 0.17 g/L, and KCl 0.37 g/L at pH 7.3) supplemented with (0.1%) l-cysteine (VWR, Solon, OH, USA) was poured over them and added to an anaerobic jar to preserve the microorganisms during transportation. All samples were processed upon arrival to the laboratory within an hour of collection. The intestinal tract was laid out, Ringers solution + (0.1%) l-cysteine sprayed over the tracts, and the mesentery, liver, heart, pancreas, and gallbladder were removed. Butchers twine was used to knot directly above and under the ceca to prevent the mixing of gut contents. The ceca were cut open, contents were removed, and then the mucosa was scrapped and added to a 15-mL centrifuge tube. Mucosal scrapings from both ceca were added to the same tube. The dissection equipment was cleaned and sterilized with 70% ethanol before scraping off the mucosa. All intestinal tracts were screened for *Salmonella* infection using MacConkey Agar.

### 2.3. Determination of Antibacterial Activity

#### 2.3.1. Screening Using Double Layer Technique

The protocol was modified from Reference [21]. Furthermore, 9 mL of de Man, Rogosa, and Sharpe (MRS) broth (Criterion, Hardy Diagnostics, CA, USA) supplemented with (0.l%) l-cysteine was added to the 15-mL centrifuge tube containing ceca mucosal scrapings, vortexed to ensure mixing, and then 10-fold serial dilutions were performed. Aliquots of 100 μL were spread onto either 1.2% MRS agar or tryptic soy agar (TSA) (1.2%) (Criterion, Hardy Diagnostics, CA, USA) supplemented with (0.5%) Yeast extract (YE) (Biobasic, NY, USA), seeded with 1% *S.* Abony NCTC 6017. One set of Petri plates was incubated aerobically, and the other set was incubated anaerobically at 37 °C for 48 h or 72 h, respectively. Those colonies that yielded inhibition zones were selected and cultured in MRS broth + (0.1%) l-cysteine for 24 h to be used for a spot-on lawn method as a first mean of screening.

#### 2.3.2. Spot-on Lawn Method

The protocol was adapted from Reference [22]. An aliquot of 3 μL of an overnight culture of isolated strains were spotted onto the surface of MRS (1.2%) agar or tryptic soy broth with yeast extract (TSBYE) (1.2%) agar pre-seeded with 1% overnight culture of *S.* Abony NCTC 6017 and incubated either aerobically or anaerobically at 37 °C for 24 h. Those strains that showed inhibition zones were selected, purified three times by streaking on their respective media, and stock solutions prepared and maintained at −20 °C and stored at −80 °C in their represented media containing 50% glycerol (*v/v*).

#### 2.3.3. Agar Well-Diffusion Method

The protocol was adapted from Reference [23]. Briefly, 25 mL of sterile MRS Agar (1.2%) was seeded with 1% *S.* Abony NCTC 6017, poured into a sterile petri dish, and allowed to solidify at room temperature for 30 min. Afterward, the wide end of a 5 mL pipette was used to create wells in the agar, and 80 μL of cell-free supernatant (CFS) or ciprofloxacin (control) was added to the wells. The plates were stored at 4 °C for 1 h to allow the CFS to diffuse through the agar and then incubated aerobically at 37 °C for 24 h. The diameter of the inhibition zone was measured. The plates were prepared in duplicate and repeated with the other indicator strains. The most active isolated strains and *S.* Typhimurium ATCC 14028 strain were chosen for the critical-dilution assay.

#### 2.3.4. Critical Dilution Assay

Antimicrobial activity was determined using the microtitration method adapted from Reference [22]. Briefly, in a 96-well flat-bottomed plate (VWR, Monroeville, PA, USA), 125 μL of CFS was added to MRS broth and used to perform two-fold serial dilutions, which were then seeded with 50 μL of 10^6^ CFU/mL of *S.* Typhimurium ATCC 14028. The plates were incubated for 24 h and absorbance at 600 nm was measured every 20 min using a Tecan Microplate Reader Spark^®^ (Grödig, Austria).

### 2.4. Identification of Isolated Strains by 16S rRNA Sequencing

The active strains were identified by 16S rRNA. Genomic DNA was extracted from the overnight culture of isolated strains using a NucleoSpin Microbial DNA kit (Macherey-Nagel, Duren, Germany) as per manufacturer’s instructions. The DNA’s concentration and purity was then determined using the Tecan NanoQuant plate (Austria) by comparing the absorbance ratio at 260 nm to 280 nm.

The 16S rRNA gene was amplified by a PCR thermal cycler (Eppendorf, Hamburg, Germany) using the universal primers 1391-R (5′-GACGGGCGGTGTGTR) and Bact-8F (5′-AGAGTTTGATCCTGGCTCAG-3′) (Millipore-Sigma, Cleveland, OH, United States), in a total volume of 50 μL [24]. The reaction mixture contained 1× PCR buffer (Invitrogen, Carlsbad, CA, USA), 1.5 mM MgCl_2_ (Invitrogen, Carlsbad, CA, USA), 0.2 mM NTPS (Invitrogen, Carlsbad, CA, USA), 1 µM of each primer, 0.2 μL of a Taq polymerase (Invitrogen), 36.3 μL of H_2_O, and 20 ng of bacterial DNA. The thermal cycler program consisted of an initial hold at 95 °C for 5 min for denaturation and polymerase activation, 30 cycles of 94 °C for 30 s, 55 °C for 30 s, 72 °C for 1 min 30 s, and a final elongation step of 5 min at 72 °C. The PCR products were then purified using a QIAquick PCR purification kit (Qiagen, Hilden, Germany) and sequenced by the Ottawa Hospital Research Institute’s DNA sequencing facility (Ottawa, ON, Canada). The resulting sequences were aligned against 16S ribosomal RNA database using the BLAST program [25]. The criterion used to identify an isolate to the species level was having an identity greater than 99% in the 16S rRNA gene sequence.

### 2.5. Whole Genome Sequencing

The whole genome sequencing library was prepared using Nextera™ DNA Flex Library Prep (Illumina; San Diego, CA, USA) as per its protocol. The prepared library was paired end sequenced (2 × 151 bp) in a 1/20 Miseq run with Illumina MiSeq platform (NuGUT Research Platform, University of Ottawa, Ottawa, ON, Canada) using a 300-bp MiSeq Reagent Kit v2 (Illumina, San Diego, CA, USA). The generated reads were de-novo assembled using the Velvet Assembler V1.0.0 incorporated in Illumina BaseSpace Sequence Hub (Illumina). Kraken2 Metagenomics V2.0.0 [26] was used to assign the taxonomy to the generated reads. The assembled contigs were annotated using Rapid Annotation using Subsystem Technology (RAST) server [27,28,29]. BAGEL4 and antiSMASH 5.0 [30] was used to screen for secondary metabolite genes in the assembled genomes. The assembled contigs were submitted to the PHAge Search Tool Enhanced Release (PHASTER) [31] for identification and annotation of the prophage sequences.

### 2.6. Safety Evaluation and In Vitro Probiotic Potential

#### 2.6.1. Antibiotic Susceptibility Test

The protocol was adapted from Reference [22]. In a 96-well flat-bottomed plate, 100 μL of twice the selected antibiotic concentration was added to 100 μL of MRS broth with (0.1%) l-cysteine and used to perform two-fold serial dilutions. The wells were then seeded with 100 μL of 10^5^ CFU/mL of the selected bioactive strains. The absorbance at 600 nm was measured using Tecan Microplate Reader Spark^®^ (Grödig, Austria) at 0 h and then incubated in the anaerobic chamber for 24 h. Then absorbance was read once again. The minimum inhibitory concentration (MIC) was determined as the lowest concentration of the antibiotic that inhibited the visible growth of the microorganism. The antibiotics tested were ampicillin, vancomycin, gentamycin, streptomycin, erythromycin, tetracycline, and chloramphenicol (All antibiotics were obtained from Alfa Aesar, Mississauga, Canada apart from gentamycin, which was from VWR, New York, NY, USA).

#### 2.6.2. Tolerance to Bile Salts and Gastric Acidity

The protocol was adapted from Reference [17]. Simulated gastric juice (SGJ) and intestinal juice (SIJ) was prepared according to the United States Pharmacopoeia (USPCCE 2004) [32]. SIJ consisted of 10 g L^−1^ pancreatin (Wards Science, St Catherines, Canada) dissolved in 0.05 mol L^−1^ KH_2_PO_4_, at pH 7·4, and SGJ consisted of 2 g L^−1^ pepsin (Wards Science, St Catherines, Canada) and 2 g L^−1^ NaCl at pH 1·5 [32]. The SIJ was used to test bile salts’ tolerance of the isolated strains, and the SGJ was used to test tolerance to pH. To the SIJ, oxgall bile salts (VWR, Mississauga, Canada) were added at varying concentrations of 0.0%, 0.1%, 0.2%, 0.3%, 0.4%, 0.6%, 0.8%, 1%, and 1.5% to mimic the physiological concentrations of the intestinal tract [17]. The SGJ was divided into centrifuge tubes with a pH of 2.6 and 4.5, the gizzard’s pH, and the crop, respectively [33]. The pH was adjusted using 5 N NaOH or 1 M HCl. All fluids were filter sterilized with a 0.45 µm filter.

A 96-well flat-bottomed plate was used to determine the tolerance to bile salts. 100 μL of intestinal juice with different bile salt concentrations was added to the wells, in triplicate, and followed by 100 μL of overnight cultures of the isolated strains. The resulting 200 μL was mixed with a multi-channel pipette. Enumerations using the drop plate method were performed at 0, 3, 6, and 24 h. The plate was incubated anaerobically at 37 °C. Similarly, the tolerance to pH was tested using 100 μL of pH-adjusted gastric juice seeded with 100 μL of overnight cultures of the isolated strains cultured for 18 h. Enumeration using the drop plate method was performed at 0 h, 15 min, 30 min, and 90 min. The controls consisted of intestinal juice or gastric juice with no digestive enzymes. The assay was performed in triplicates.

### 2.7. Statistical Analysis

All experiments were carried out in triplicate. Results were log-transformed. Graph pad Prism 8 was used to perform statistical analysis. A *p*-value < 0.05 was considered to be significant. One-way ANOVA was used to compare control and treatment samples in the bile salt tolerance test. A paired *t*-test was used to compare control to treatment samples in the pH tolerance test. Dunnett’s multiple comparisons test was used to analyze further results obtained from one-way ANOVA.

## 3. Results

### 3.1. Screening and Isolation of Bioactive Strains and Characterization of Their Inhibitory Activity

**Figure 1:**
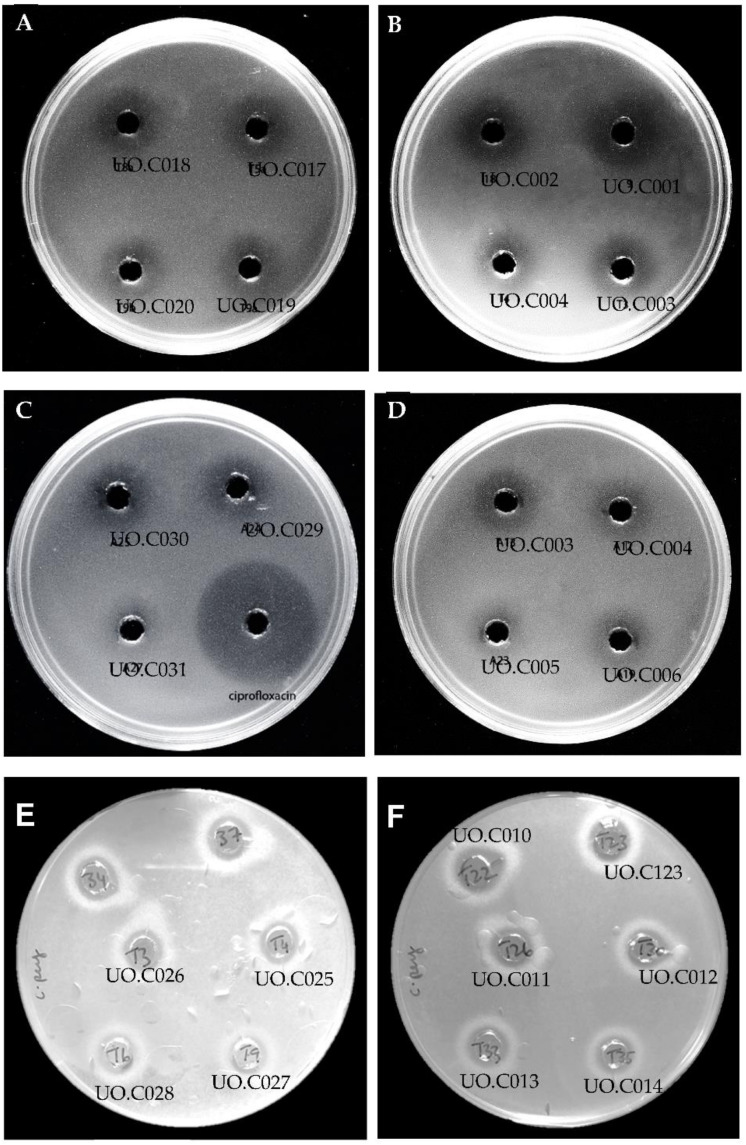
Agar well diffusion assay illustrating the growth inhibition of *Salmonella* Typhimurium ATCC 14028 (**A**–**D**) and *Clostridium perfringens* ATCC 13124 (**E**,**F**) by cell-free supernatants extracted from the isolated strains.

**Figure 2:**
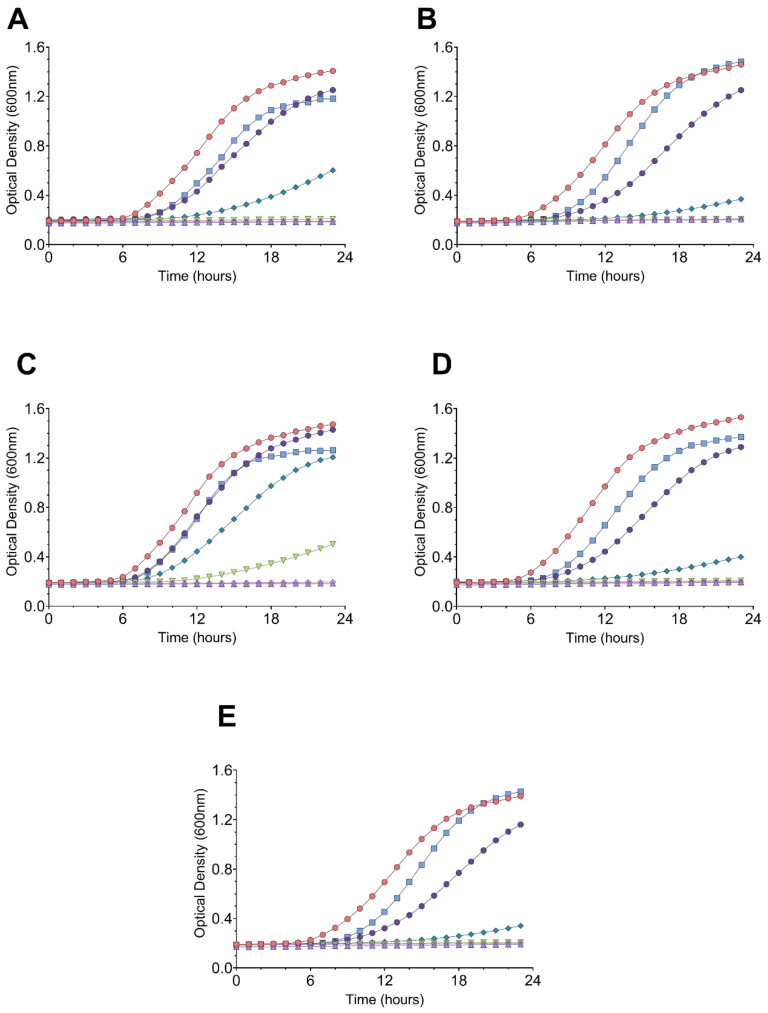
Dose-response growth inhibition of *Salmonella* Typhimurium ATCC 14028 by cell-free supernatants from strains UO.C025 (**A**), UO.C027 (**B**), UO.C031 (**C**), UO.C003 (**D**), and UO.C018 (**E**). Tested concentrations are 1× (purple triangle), 1/2 (magenta diamond), 1/4 (triangle), 1/8 (diamond), 1/16 (dark purple circle), 1/32 (blue square), and 0 (negative control, red circle).

A total of 290 colonies were initially selected based on inhibition zones, from all four intestinal tracts obtained from a local slaughterhouse. Screening for the presence of *Salmonella* was carried out on MacConkey agar plates, and all the tested intestines were most likely free from *Salmonella* infection in view of no enrichment step that was followed. The samples were first screened by a spot-on lawn method (data not shown). Of the initial 290 colonies, the number of active isolated strains were narrowed down to 72 isolates that showed activity against *S.* Abony NCTC 6017. This step was followed by another round of screening using agar well diffusion, which narrowed down our active colonies from 72 to 55 isolates showing strong inhibition of *S.* Abony NCTC 6017. Next, we tested the cell-free supernatants (CFS) of the 55 isolates against *C. perfringens* ATCC 13124, *S.* Abony NCTC 6017, *S. enterica* serovar Typhimurium ATCC 14028, *S. enterica* serovar Choleraesuis ATCC 10708, and *E. coli* ATCC 25922. Figure 1 illustrates the inhibition of indicator strains by some selected strains’ CFS, while the inhibitions’ diameter of active strains is summarized in Table 1. As shown in Figure 2, the CFS of colonies UO.C025, UO.C027, UO.C031, UO.C003, and UO.C018 induced a dose-dependent inhibition of *S.* Typhimurium ATCC 14028 over 24 h of incubation. The CFS of the five strains had similar antimicrobial potency and completely inhibited the growth of *Salmonella* at 1×, ½×, and ¼×, while it induced partial inhibition at higher dilutions.

### 3.2. Molecular Identification of Isolated Bioactive Strains

The 16S rRNA of the most active strains (31) was sequenced. The isolates belonged to *Ligilactobacillus salivarius* (*n* = 19), *Ligilactobacillus agilis* (*n* = 1), *Lactobacillus johnsonii* (*n* = 7), *Lactobacillus kitasatonis* (*n* = 1), *Lactobacillus* sp. (*n* = 2), and uncultured bacterium (*n* = 1). Among the identified strains, UO.C003 was identified as *Ligilactobacillus salivarius*, UO.C018 as *Ligilactobacillus agilis*, UO.C025 as *Lactobacillus kitasatonis*, and UO.C027 as *Ligilactobacillus salivarius* (Table 2). The molecular identification of the strain UO.C031 revealed a new unclassified bacterium with partial similarity to *Lactobacillus gallinarum*.

### 3.3. Draft Whole-Genome Sequence and Bacteriocin Genome-Mining of the Selected Candidates

Kraken2 confirmed the 16S rRNA-based identity of UO.C003, UO.C018, and UO.C027 where 92%, 73%, and 93% of the generated reads were assigned to *L. salivarius*, *L. agilis*, and *L. salivarius*, respectively. The generated reads of UO.C025 and UO.C031 were assigned to the genus *Lactobacillus* with 21% and 23% of the reads with no hits in the database. Table 3 summarizes the genomic information of these strains and their closest phylogenetic neighbor generated by RAST. Table 4 shows, in detail, all the bacteriocin genes that were identified, their location, and their similarity to BLAST search using both BAGEL4 and antiSMASH. BAGEL4 was able to identify the presence of bacteriocin genes in four of the five isolated strains with enterolysin A being the commonly identified bacteriocin gene. *Lactobacillus kitasatonis* also had Helveticin-J genes, and *Ligilactobacillus salivarius* had salivaricin_P_chain_b genes. Likewise, antiSMASH identified lanthipeptide genes in *Lactobacillus kitasatonis* and Linocin_M18 genes in *Lactobacillus gallinarum.* The *Ligilactobacillus salivarius* strain had salivaricin CRL1328 α peptide/salivaricin CRL1328 β peptide with 75% of genes showing a similarity. The PHAge Search Tool Enhanced Release (PHASTER) (Arndt et al., 2016) identified a number of prophage regions in the sequenced genome that ranged from 0 to 3 regions per genome, as summarized in Table 5.

### 3.4. Probiotic Potential of Bioactive Strains

The breakpoints cut-off values were determined from the European Food Safety Authority (EFSA) panel on additives and products or substances used in animal feed (FEEDAP) [34]. *L. agilis* UO.C018 and *L. kitasatonis* UO.C025 breakpoint cut-off values were assumed to be the same as *L. salivarius*. All four are facultative homofermentative species. All tested strains showed susceptibility to ampicillin, with *L. kitasatonis* UO.C025 and uncultured bacterium UO.C031 showing susceptibility to vancomycin as well. All isolates showed resistance to gentamycin, streptomycin, erythromycin, tetracycline, and chloramphenicol (Table 6).

**Figure 3:**
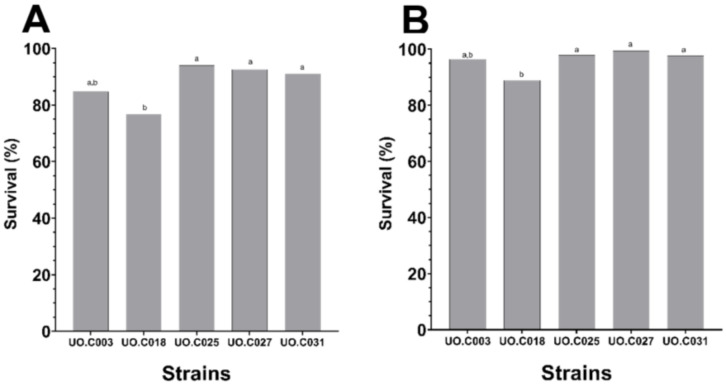
Percentage of survival of selected bioactive strains under acidic pH 2.6 (Gizzard, **A**) and pH 4.5 (Crop, **B**) in a simulated gastric juice. a and b labels the strains that exhibit significant differences in their survival at each pH.

**Figure 4:**
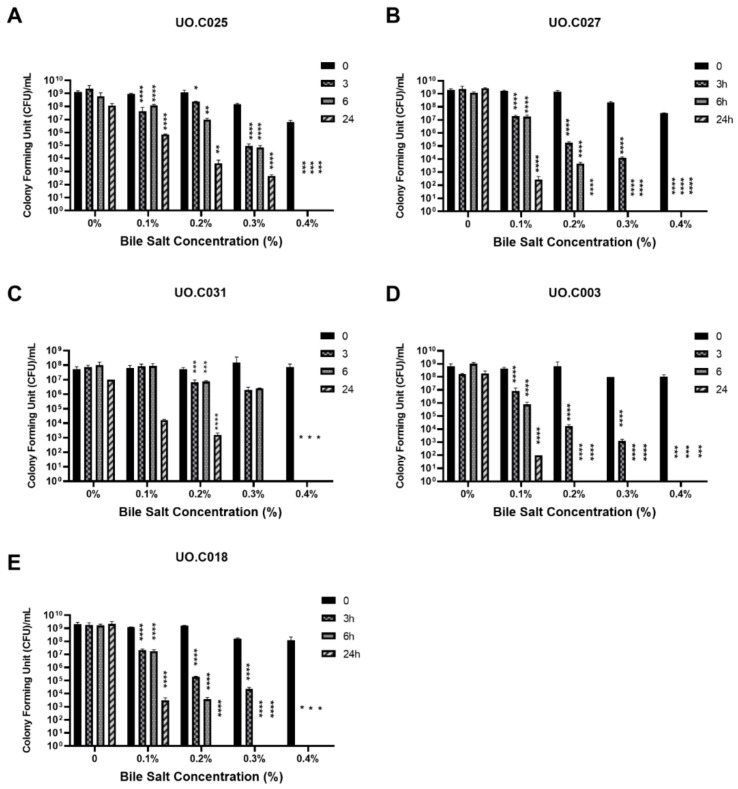
Isolated strains tested for bile salt tolerance in simulated intestinal juice at times 0, 3, 6, and 24 h. One-way ANOVA followed by Dunnett’s multiple comparison test were used for statistical comparison of the difference between each time point and the zero time. * *p* < 0.05, ** *p* < 0.01, *** *p* < 0.001, **** *p* < 0.0001 as compared to the zero time of each concentration.

The selected strains were also subjected to acidic pH and bile salts under simulated gastric intestinal and juices to determine the viability of our isolated strains under harsh gastrointestinal conditions. For the pH tolerance test, simulated gastric juice with a pH of 2.6 and 4.5 was selected to represent the physiological pH of the gizzard and the crop segments, respectively. The selected strains had a high survival rate at both pH 4.5 and 2.6 with a respective range of 88.97%–99.33% and 76.80%–93.98% (Figure 3). The strains *L. kitasatonis* UO.C025, *L. salivarius* UO.C027, and uncultured bacterium UO.C031 exhibited the highest survival rate, while *L. agilis* UO.C018 was the most sensitive to the two tested pH values. For the bile salts’ tolerance test, varying concentrations of oxgall bile salts were added to the simulated intestinal juice, containing pancreatin. The bile salts concentrations tested included 0.1%, 0.2%, 0.3%, 0.4%, 0.6%, 0.8%, 1%, and 1.5%. The lowest concentration to show growth after at least 3 h was 0.3% bile salts, while no growth of tested strains was observed at higher concentrations. The difference between the control and the treated samples was statistically significant (*p*-value < 0.05, Figure 4).

## 4. Discussion

Probiotics have been shown to modulate the immune system, increase the proliferation of beneficial commensal bacteria, increase nutrient absorption, and, hence, increase feed conversion efficiency, and increase body weight [12]. For some probiotics, such as Lactobacilli, these beneficial properties are due to the production of bacteriocins and short-chain fatty acids. Lactobacilli and other anaerobic bacteria are found to adhere to the epithelium of the intestinal tract, which is also abundant with *Bifidobacterium* sp., *Enterococcus faecium*, and *Pediococcus* spp., with the ceca having the highest concentration of anaerobic bacteria [35]. FAO and WHO recommend that a probiotic be able to survive the passage, adhere, and multiply through the GIT, be a Gram-positive organism, show measurable health benefits, and have a defined dosage duration [36,37]. In this study, we aimed to isolate potential probiotic bacterial strains from the cecum mucosa from broiler chickens that could reduce *Salmonella enterica and C. perfringens*, which are important foodborne pathogens. We chose to target the mucosa of the intestinal tract rather than the lumen contents because the commensal strains are already ideally suited to that specific environment via adhesion and competitive exclusion, and, therefore, have a head start at potential probiotic properties [38].

To accelerate the isolation and screening of potential probiotic strains, instead of allowing the colonies to grow on MRS agar plates for 24–48 h as per a standard protocol, we adapted the protocol to simultaneously allow the growth of colonies while they exhibit inhibition zones on the agar plates. A total of 290 presumably active isolates, that are aerobic and anaerobic, from four intestines were selected based on the presence of inhibition zones in the initial double agar method. These colonies were further screened using the spot-on lawn method and, then, the agar well diffusion assay, resulting in 55 inhibitory strains against *Salmonella*. Additionally, dose-dependent inhibition of *Salmonella* over 24 h was observed in all the tested strains, which might be attributed to the presence of short-chain fatty acids (lactic acid, propionate, butyrate, and acetate), hydrogen peroxide, or bacteriocin-like compounds. For instance, selected bioactive strains belong to the lactobacilli group, members of the lactic acid bacteria, a broadly defined group characterized by lactic acid production as a sole or main end product of carbohydrate fermentation [39].

The predominant identity of the isolated strains was from *L. salivarius,* followed by *L. johnsonii*. We also isolated and identified other lactobacilli strains belonging *to L. agilis*, *L. kitasatonis*, *Lactobacillus* sp., and a new uncultured bacterium. This is consistent with findings that the *L. acidophilus* group (*L. crispatus*, *L. gallinarum*, and *L. johnsonii*), *L. agilis*, *L. salivarius*, and *L. reuteri* are commonly present Lactobacilli in chicken intestines [40]. From among the *L. acidophilus* group, it was found that *L. salivarius* was the predominant species among the intestinal microbiota of young chickens, which is consistent with what we found from our isolation results [40]. *L. kitasatonis* was originally isolated from the intestinal tract of chickens in Japan in 2003 [41]. Since then, *L. kitasatonis* has been isolated from dogs in Japan [42], geese feces in Poland [43], and pig feces in Italy [44]. Different species are found along the chicken intestinal tract. Lactobacilli species are found in high concentrations in the upper GIT but also in the ileum than cecum [38]. It has also been reported that *L. salivarius* and *L. johnsonii* are found in a higher percentage in the ileal mucosa when compared to cecal mucosa or cecal lumen, but there was no statistical difference between them [38]. The same researchers found *L. salivarius* to be a predominant species along with the entire GIT, which is consistent with other reports that, in 36-days-old chickens, *L. salivarius* has a higher percentage of being isolated from both the ileum and cecum [38].

All isolated strains showed susceptibility to ampicillin but resistance to vancomycin, gentamycin, streptomycin, erythromycin, tetracycline, and chloramphenicol. Lactobacilli are considered non-pathogenic and are widely used as probiotics and starter cultures for various foods, and are supported by a long history of safe usage [45]. Despite their safety status, many *Lactobacillus* species have been reported as intrinsically resistant to antibiotics, such as vancomycin, gentamycin, kanamycin, streptomycin, and ciprofloxacin, but susceptible to penicillin and β-lactams [45]. Our whole genome sequencing (WGS) results showed that our isolated strains have the capability to produce bacteriocins and lack plasmids, toxins, adhesion toxins, and transposable elements, indicating that their resistance profiles are chromosome-mediated.

Resistance to harsh gastrointestinal conditions is important during probiotic selection. Our selected strains exhibited a high survival rate at acidic conditions mimicking the physiological pH of crop and gizzard, indicating their suitability as potential probiotics since they would be able to survive passage through the gastrointestinal tract (GIT) and attach to the mucosa via competitive exclusion (the strains were originally isolated from the mucosa of the ceca). Our results showed superior results to those reported by Reference [46], whose *L. salivarius* and *L. kitasatonis* strains could not survive the acid tolerance test after one-hour incubation. Our selected strains were able to survive the tested bile salt concentration of 0.3% for at least 3 h. Similar results were obtained by Reference [46], who observed that only a single *L. kitasatonis* strain was able to survive growth in 0.3% oxgall bile salts. The actual bile salt concentration in chicken duodenum, jejunum, and cecum is 1.75 mg/mL, 7 mg/mL, and 0.085 mg/mL, respectively [47]. Our strains were able to survive at the tested oxgall concentration of 3 mg/mL, indicating that they would survive passage to the duodenum and ceca if well formulated.

## 5. Conclusions

The present study provides evidence that lactobacilli strains isolated from chicken caecal mucosa harbor bacteriocin genes and produce inhibitory substances against *Salmonella enterica* and *Clostridium perfringens*. While *L. salivarius* UO.C003, *L. agilis* UO.C018, *L. kitasatonis* UO.C025, *L. salivarius* UO.C027, and a new uncultured bacterium presented some interesting probiotic features. A further investigation is required toward their application as novel probiotic strains in the poultry industry.

## Figures and Tables

**Table 1:** Determining the diameter of indicator strain inhibition by cell-free supernatants extracted from bioactive isolated strains in this study.

Strains ID	Inhibition Zone (mm)				
	*S.* Abony NCTC 6017	*S.* Typhimurium ATCC 14028	*S.* Choleraesuis ATCC 10708	*E. coli* ATCC 25922	*C. perfringens* ATCC 13124
UO.C001	15	12	14	10	10
UO.C002	14	15	11	10	12
UO.C003	13	16	15	10	12
UO.C004	12	15	10	10	11
UO.C005	13	15	10	6	11
UO.C006	13	15	11	10	11
UO.C007	14	14	13	10	14
UO.C008	11	15	10	10	12
UO.C009	10	14	13	9	11
UO.C010	15	12	11	10	12
UO.C011	14	14	11	10	12
UO.C012	15	13	10	6	10
UO.C013	14	15	14	9	11
UO.C014	14	14	10	9	11
UO.C015	13	15	13	7	10
UO.C016	13	15	13	7	12
UO.C017	15	14	10	7	11
UO.C018	13	15	10	8	10
UO.C019	12	13	10	7	11
UO.C020	13	15	10	8	11
UO.C021	12	15	15	-	14
UO.C022	-	12	15	-	13
UO.C023	-	14	14	0.7	13
UO.C024	-	12	14	-	13
UO.C025	11	13	14	-	13
UO.C026	-	12	17	-	14
UO.C027	11	13	15	0.9	14
UO.C028	-	12	14	-	14
UO.C029	-	16	13	0.8	14
UO.C030	-	14	14	-	14
UO.C031	-	14	13	-	14
UO.C112	10	13	10	7	9
UO.C121	10	12	9	7	8
UO.C123	11	13	12	6	11
UO.C124	12	16	-	10	7
UO.C134	10	19	-	10	8
UO.C137	11	15	13	10	10
UO.CA05	-	14	12	-	12
UO.CA22	-	13	10	-	13

**Table 2:** Molecular identification of isolated bioactive strains by 16S rRNA sequencing. Strains in bold represent the five most potent strains that were further characterized for their probiotic potential.

Chicken	Location/Type	Anatomy	Sample Id	Identity
**#3**	mucosal/aerobic	ceca	UO.C001	*Ligilactobacillus salivarius*
**#3**	mucosal/aerobic	ceca	UO.C002	*Ligilactobacillus salivarius*
**#4**	mucosal/aerobic	ceca	**UO.C003**	***Ligilactobacillus salivarius***
**#4**	mucosal/aerobic	ceca	UO.C004	*Ligilactobacillus salivarius*
**#4**	mucosal/aerobic	ceca	UO.C005	*Lactobacillus johnsonii*
**#4**	mucosal/aerobic	ceca	UO.C006	*Lactobacillus* sp.
**#4**	mucosal/aerobic	ceca	UO.C007	*Ligilactobacillus salivarius*
**#4**	mucosal/aerobic	ceca	UO.C008	*Ligilactobacillus salivarius*
**#4**	mucosal/aerobic	ceca	UO.C009	*Ligilactobacillus salivarius*
**#4**	mucosal/aerobic	ceca	UO.C010	*Ligilactobacillus salivarius*
**#4**	mucosal/aerobic	ceca	UO.C011	*Ligilactobacillus salivarius*
**#4**	mucosal/aerobic	ceca	UO.C012	*Lactobacillus* sp.
**#4**	mucosal/aerobic	ceca	UO.C013	*Ligilactobacillus salivarius*
**#4**	mucosal/aerobic	ceca	UO.C014	*Lactobacillus johnsonii*
**#4**	mucosal/aerobic	ceca	UO.C015	*Ligilactobacillus salivarius*
**#4**	mucosal/aerobic	ceca	UO.C016	*Ligilactobacillus salivarius*
**#1**	mucosal/aerobic	ceca	UO.C017	*Ligilactobacillus salivarius*
**#1**	mucosal/aerobic	ceca	**UO.C018**	***Ligilactobacillus agilis***
**#1**	mucosal/aerobic	ceca	UO.C019	*Ligilactobacillus salivarius*
**#1**	mucosal/aerobic	ceca	UO.C020	*Ligilactobacillus salivarius*
**#4**	mucosal/anaerobic	ceca	UO.C021	*Lactobacillus johnsonii*
**#4**	mucosal/anaerobic	ceca	UO.C022	*Lactobacillus johnsonii*
**#4**	mucosal/anaerobic	ceca	UO.C023	*Ligilactobacillus salivarius*
**#4**	mucosal/anaerobic	ceca	UO.C024	*Lactobacillus johnsonii*
**#4**	mucosal/anaerobic	ceca	**UO.C025**	***Lactobacillus kitasatonis***
**#4**	mucosal/anaerobic	ceca	UO.C026	*Ligilactobacillus salivarius*
**#4**	mucosal/anaerobic	ceca	**UO.C027**	***Ligilactobacillus salivarius***
**#3**	mucosal/anaerobic	ceca	UO.C028	*Lactobacillus johnsonii*
**#3**	mucosal/anaerobic	ceca	UO.C029	*Ligilactobacillus salivarius*
**#3**	mucosal/anaerobic	ceca	UO.C030	*Lactobacillus johnsonii*
**#3**	mucosal/anaerobic	ceca	**UO.C031**	**UNCULTURED bacterium**

**Table 3:** Whole genome draft sequence overview as annotated by Rapid Annotation using Subsystem Technology (RAST) server.

Strains	16S rRNA Identity	Genome Size (bp)	GC Content	Contig	N50	L50	Number of Subsystems	Number of Coding Sequences	Number of RNAs	Closest Neighbour
**UO.C003**	*L. salivarius*	2,024,427	32.7	100	176765	4	211	1994	76	*L. salivarius* ATCC 11741
**UO.C018**	*L. agilis*	2,345,855	41.0	110	116853	7	214	2346	92	*L. salivarius* ATCC 11741
**UO.C025**	*L. kitasatonis*	1,975,848	37.4	163	47569	16	189	2077	58	*L. delbrueckii* subsp. *bulgaricus* ATCC 11842
**UO.C027**	*L. salivarius*	1,848,796	32.8	76	261149	2	212	1087	70	*L. salivarius* ATCC 11741
**UO.C031**	*L. gallinarum*	2,014,858	36.5	145	51519	15	192	2092	74	*L. acidophilus* NCFM

**Table 4:** Overview of all the bacteriocin genes that were identified, their location, and their similarity to BLAST search using both antiSMASH and BAGEL4.

**Strain**	**BLAST Identity**	**Identified Bacteriocin**	**Region**	**From**	**To**	**Similarity**	**Database**
UO.C003	*L. salivarius*	salivaricin CRL1328 α peptide/ salivaricin CRL1328 β peptide	28.1	27,593	46,844	75%	antiSMASH
UO.C025	*L. kitasatonis*	Lanthipeptide	29.1	1	19,666		antiSMASH
UO.C031	*L. gallinarum*	Linocin_M18	15.1	33,984	44,931		antiSMASH
**Strain**	**BLAST Identity**	**Identified Bacteriocin**	**NODE**	**From**	**To**	**Blast Result**	**Database**
UO.C003	*L. salivarius*	63.3, Enterolysin A	8	149,321	169,768	49%	BAGEL4
		153.2, MR10B	15	3548	19,814	85.33%	
		213.2, Salivaricin_P_chain_b	83	18,395	51,832	58.54%	
		E64.3, Enterolysin A	1	165,542	186,031	38.93%	
UO.C025	*L. kitasatonis*	70.3, Helveticin J	60	3635	15,615	37.15%	BAGEL4
		62.3, Enterolysin A	93	1	14,236	62.73%	
		64.3, Enterolysin A	93	11,342	23,788	81.22%	
		6.3 Helveticin J	190	39,656	56,375	90.20%	

**Table 5:** Overview of prophage regions identified in the draft genomes of the selected bioactive strains by the PHAge Search Tool Enhanced Release (PHASTER) (Arndt et al., 2016).

Strain	Number of Prophage Regions	Region Length	Completeness	Most Common Phage
UO.C025	0			
UO.C027	1	21.3 kb (16 protein)	incomplete	PHAGE_Staphy_SPbeta_like_NC_029119
UO.C031	2	23.5 kb (35 protein)	incomplete	PHAGE_Lactob_Lj965_NC_005355
		28.1 kb (32 protein)	incomplete	PHAGE_Lactob_LBR48_NC_027990
UO.C003	3	59.5 kb (63 protein)	intact	PHAGE_Lactob_CL1_NC_028888
		25.3 kb (26 protein)	incomplete	PHAGE_Lactob_PLE2_NC_031036
		16.9 kb (20 protein)	questionable	PHAGE_Paenib_Shelly_NC_041909
UO.C018	1	57.2 kb (70 proteins)	intact	PHAGE_Lactob_Sha1_NC_019489

**Table 6:** Minimum inhibitory concentration (MIC in μg/mL) and interpretation of selected strains.

Antibiotics	Breakpoints	UO.C003		UO.C018		UO.C025		UO.C027		UO.C031	
		MIC	Susceptibility	MIC	Susceptibility	MIC	Susceptibility	MIC	Susceptibility	MIC	Susceptibility
Ampicillin	4	2	S	2	S	2	S	4	S	2	S
Vancomycin	*n*.r	>32	R	>32	R	4	S	>32	R	4	S
Gentamycin	16	>32	R	>32	R	>32	R	>32	R	>32	R
Streptomycin	64	>32	R	>32	R	128	R	>32	R	128	R
Erythromycin	1	2	R	4	R	1	S	4	R	2	R
Tetracycline	8	>32	R	>32	R	>32	R	>32	R	8	S
Chloramphenicol	4	16	R	16	R	16	R	>32	R	16	R

## Data Availability

The whole genome sequence of the five tested strains will be deposited at GenBank under the BioProject accession number PRJNA685183.
